# Ablation of Tumor Necrosis Factor Is Associated with Decreased Inflammation and Alterations of the Microbiota in a Mouse Model of Inflammatory Bowel Disease

**DOI:** 10.1371/journal.pone.0119441

**Published:** 2015-03-16

**Authors:** Yava L. Jones-Hall, Ariangela Kozik, Cindy Nakatsu

**Affiliations:** 1 Department of Comparative Pathobiology, Purdue University, West Lafayette, Indiana, United States of America; 2 Department of Agronomy, Purdue University, West Lafayette, Indiana, United States of America; 3 Purdue University Interdisciplinary Life Science Program (PULSe), West Lafayette, Indiana, United States of America; Charité, Campus Benjamin Franklin, GERMANY

## Abstract

Inflammatory bowel disease (IBD) is associated with prolonged, excess secretions of Tumor Necrosis Factor (TNF). Many patients with IBD have successful management of IBD symptoms by blocking TNF secretion or signaling. However, some patients are non-responsive to this therapy, eventually become refractory to therapy, or Alterations in the microbiota that are associated with the lack of TNF could be a contributing cause of this therapeutic insufficiency seen in some patients. Here we use wildtype (WT) and mice lacking *Tnf* (*Tnf* ^-/-^) in an acute TNBS colitis model to investigate the role of TNF in colitis and how its presence or absence affects the colonic microbiota. As expected, *Tnf* ^-/-^ had less severe inflammation than WT mice. Microbiome analysis revealed significant *Tnf* dependent-differences in alpha and beta diversity. There were also notable differences in many species that were also primarily *Tnf* dependent. Taken together, our data indicates that TNF contributes significantly to the inflammation and microbiotal alterations in that occur in IBD.

## Introduction

Inflammatory bowel disease (IBD) consists of two inflammatory disorders, Crohn’s disease (CD) and Ulcerative Colitis (UC), both of which are characterized by lifelong and repeated bouts of inflammation and healing in the gastrointestinal (GI) tract [1]. The pathogenesis of these diseases has not been fully elucidated, however, it is generally accepted that disease develops in genetically susceptible individuals that have hyper-immune responsiveness to their intestinal microbiota [2–5]. The mucin layer covering the intestinal epithelia is the first line of defense in preventing the attachment and invasion of luminal bacteria to the intestinal epithelia [6,7]. IBD patients have deficient or aberrant mucin [8], which likely leads to displacement of luminal bacteria to the submucosal compartment and the initiation of inflammation [9]. Epithelial cells form the next barrier of protection of the submucosal compartment from luminal bacteria. Increased translocation of bacteria has been associated with increased risk of developing IBD which is thought to be due primarily to dysregulation of the epithelial barrier (reviewed in: [10]). In IBD patients many pro-inflammatory cytokines, such as tumor necrosis factor (TNF), interferon gamma (INFγ), interleukin (IL)-6, IL-17 and members of the IL-12 family, are produced in excess in response to the translocated intestinal microbiota and these responses have been shown to be instrumental to the progression of disease [11–13]. Confounding this exaggerated inflammatory response to bacteria in the submucosal compartment is the inhibition of epithelial repair by TNF-induced upregulation of apoptosis (reviewed in: [14]). Here, we discuss and provide data showing how TNF induced inflammation and microbial alterations contribute to the progression of IBD in a mouse model.

The human gut is the largest reservoir of microbes in the body. Many studies have indicated that the gut microbiota plays an active and integral role in maintaining host health [15,16]. The Human Microbiome Project has allowed us to obtain an overview of the healthy gut microbial community that is comprised of microbes belonging to a limited number of phyla but includes hundreds to thousands of species [17]. Perturbation of the gut microbiota contributes to a myriad of disease conditions, such as obesity, diabetes, metabolic syndrome, inflammation, as well as certain cancers [18]. In IBD, dysbiosis in the gut microbiota may contribute to the development of CD (and UC) in susceptible individuals emerged [19–24]. IBD-related microbial dysbiosis is characterized by a decrease in overall alpha diversity, decrease in the abundances of members of the phyla Firmicutes and Bacteroidetes, and increases in Gammaproteobacteria (reviewed in [24]). The only bacterial genus that is significantly higher in adult and pediatric IBD patients is the *Escherichia-Shigella* group [25,26]. In humans, studies are confounded by environmental and behavioral variables (e.g., smoking, antibiotic use) [26], thus model animal studies are best suited to examine the interactions between the gut microbiota and disease in order to elucidate the potential role these microbes play in IBD pathogenesis.

TNF is a pleiotropic cytokine, considered to be a master regulator of cytokine production. This cytokine is elevated in both the serum and mucosa of IBD patients [27–30]. The current, and arguably one of the most effective treatments for CD, is the use of TNF functional inhibitor drugs [31,32]; however, this treatment can cause adverse reactions [33–36], has a relatively large percentage of incomplete or non-responders and cannot be used in areas where certain infections (i.e., tuberculosis [37]) are common. The underlying reason for non-responders to anti-TNF therapy can be varied, but to date a definitive reason has not been defined. Thus, more focused therapeutic strategies are needed.

Despite intense study, the underlying etiology and mechanism(s) of pathogenesis of CD remain to be fully elucidated. Altered microbiota and excessive inflammation are two major components that contribute to the development and progression of CD and studies detailing changes in the microbiome that occur as disease progresses from acute colitis to chronic colitis are limited. We hypothesize that neutralization of TNF induces alterations in the intestinal microbiota that correlate with reduced inflammation. In this study, we use the trinotrobenzene sulfonic acid (TNBS) colitis mouse model of CD [38,39] to evaluate the colonic microbiota in healthy and colitic animals that produce *Tnf* or lack *Tnf* in order to determine how TNF suppression correlates with alterations in the microbiota and decreased inflammation. Our long term goal is to identify specific microbial communities that can be manipulated, in addition to TNF suppression, to increase the effectiveness of the anti-TNF therapeutic protocol.

## Materials and Methods

### Mice

All colitis experiments with animals in this study were approved by the Purdue Animal Care and Use Committee. Six to eight week old, female B6.129S mice with a homozygous deletion of the *Tnf* gene (*Tnf* ^*-/-*^ mice) [40] and control female B6.129S wildtype (WT) mice were obtained from The Jackson Laboratory (Bar Harbor, ME) and subsequently bred and housed at Purdue University. Mice were maintained under specific pathogen free conditions with controlled temperature (21–23°C) and 12/12-hour light/dark cycles. All mice were fed standard mouse chow. Mice were caged separately in 4 groups based on genotype (WT vs *Tnf* ^*-/-*^) and treatment (TNBS vs SHAM).

### Acute Colitis

Induction of TNBS colitis was performed according to the protocol of Wirtz *et al*. with minor modifications [41]. Five mice per treatment were tested and the experiment was performed twice resulting in 10 mice per treatment for a total of 40 mice. On day 0, animals were anesthetized with isopropanol and a 1.5 x 1.5 cm field of the skin on the back between the shoulders was shaved. One hundred μl of the TNBS (Sigma-Aldrich) sensitization solution (acetone and olive oil in a 4:1 volume was mixed rigorously by vortexing; four volumes of acetone/olive oil solution was mixed with 1 volume of 5% TNBS solution to obtain 1% w/v) was applied to the shaved area. Control mice (SHAM) were treated with sensitization solution without TNBS. On day 7, mice were anesthetized as previously stated and, using a plastic feeding gavage needle affixed to a 1-ml syringe, 100 μl of the TNBS intra-rectal (IR) solution (1 volume of 5% w/v TNBS solution mixed with 1 volume of absolute ethanol) was slowly instilled into the colon. The mice were kept with the head down in a vertical position for 60 seconds to ensure that the solution stayed in the colon. As a control (SHAM), the same volume of 50% ethanol in PBS solution was administered to additional mice. Fecal samples for microbial community analysis were collected and snap frozen on days 0 and 10 and stored at -80°C until analyzed.

### Histological Assessment of Colitis

Body weights were recorded every other day and were reported as percentage of weight loss from initial body weight. Necropsy was performed 3 days post IR injection and tissues were harvested. At necropsy, mice were euthanized by an overdose of CO2 followed by cervical dislocation. The colons were removed, opened and washed with PBS. The colons were cut in half longitudinally and 4 centimeters of one-half of the colon was fixed in 10% buffered formalin for histological examination. The remaining one-half of the colon was divided into two, 2-cm segments, which were snap frozen in liquid nitrogen and stored at -80°C. Fixed sections of colonic tissues were embedded in paraffin, cut into 6-*μ*m sections, and stained with hematoxylin and eosin (H&E) for histological analysis via light microscopy. The degree of inflammation in cross sections of the colon was assessed semi-quantitatively by an experienced pathologist, blinded to treatment allocation, as previously described [42] with minor modifications. Colons were evaluated beginning at the colorectal junction and proceeding adorally 4-cm to the middle colon. Briefly, the severity of the leukocytic infiltrate in the mucosa was subjectively assessed as none, mild, moderate or severe (0–3); and the distribution was evaluated and denoted as focal/locally extensive, multifocal, or diffuse (1–3); the distribution of erosion/ulceration was assessed as none, focal, multifocal or diffuse (0–3); necrosis was assessed as none, mild, moderate or severe (0–3). Total disease score ranges from 0 (assigned criterion not noted) to a maximum of 12 points based upon summation of each assigned criterion.

### DNA extraction

Total genomic DNA was extracted from each fecal sample (50 mg) using the Fast DNA Soil Spin kit (Q-BIO 101, Calsbad, CA) according to the manufacturer’s instructions. DNA concentration was determined using a NanoDrop 3300 (Thermo Scientific, Wilmington, DE) fluorospectrometer and quality was assessed using a NanoDrop 1000 spectrometry (260/280 OD ratio) as well as agarose gel electrophoresis.

### Sequencing and Sequence Analysis

MiSeq Illumina 2x250 paired end sequencing was used to determine fecal bacterial community composition in the first and last fecal samples collected from each mouse during the acute testing period. Primers that amplify the V3–V4 region of the 16S rRNA gene (forward TAC GGR AGG CAG CAG and reverse CTA CCR GGG TAT CTA ATC C primers) were used to take advantage of primer accuracy and coverage of phylogenetic information that has been determined for short sequencing reads [43,44]. Multiple samples were run and differentiated using a combination of 8-bp tagged forward primer and 8-bp tagged reverse primers following the manufacturer’s suggested step out protocol (Illumina). Unincorporated primers and nucleotides were separated from PCR amplicons using Agencourt AMPure XP kit (Beckman Coulter). PCR was performed using Q5 High Fidelity DNA Polymerase (New England Biolabs) to minimize error rate during polymerization (100 times lower than Taq polymerase). Purified amplicons were quantified by fluorometry after staining using the Quantifluor dsDNA Assay Kit (Promega). Amplicons from each sample were combined in equimolar quantities and sent to the Purdue Genomics facilities for sequencing using a MiSeq instrument (Illumina).

Primer tags and low quality sequence reads were first removed using Illumina software then paired end reads were merged using Panda software [45]. Sequences were analyzed using the QIIME pipeline version 1.8 [46]. The “pick open reference OTU” option with default variables and the Greengenes data set (version 13_5) were used to assign taxonomy to the representative OTU sequences [47]. All subsequent comparisons were performed using equivalent numbers of taxa (based lowest number of sequences obtained from a single sample) per sample that were chosen by rarefaction. Good’s coverage was used to obtain an estimation of sequence coverage of the communities used in these analyses. Rarefied analysis of alpha diversity richness indices (Chao1, observed species) and phylogenetic diversity (PD whole tree indices) were calculated to compare microbiota community diversity within each sample. Beta diversity comparisons between communities were made using phylogenetic distances, unweighted and weighted Unifrac [48] as well as non-phylogenetic distance analysis using Bray Curtis and binary Euclidean distances.

### Statistical analysis

Comparisons of histological scores were statistically analyzed using the nonparametric Mann-Whitney U test. All data in bar graph or dot plot formats are expressed as mean ± S.E.M. Differences were considered statistically significant at P <0.05. For the microbiome data, Krustral Wallis analysis (non-parametric equivalent to ANOVA) was used for an overall comparison of average proportions of each taxa level (phyla, class, order, family and genera) in mice fecal samples collected on day 0 (pre-colitis) and day 10 (post-colitis) of the experiments. Mann-Whitney U test was used to determine significant differences between treatments of day 10 samples. It was also used to compare day 0 and day 10 samples within treatments. The change in bacterial genera proportions was calculated by subtracting day 0 values from day 10) and significant differences were determined using the Mann-Whitney U test. All basic statistics were performed using the Paleontological Statistics package version 3.01 (PAST software, http://folk.uio.no/ohammer/past/index.html). Significant differences in beta diversity between communities were determined using PERMANOVA a non-parametric multivariate statistics [49] and PERMDISP (permutational analysis of multivariate dispersions) [50] to ensure significant differences were not due to differences in dispersion; both programs are available in the QIIME pipeline. Canonical correspondence analysis (CCA) [51] in the PC-ORD software package (Gleneden Beach, OR) was used to determine associations between the colitis scores and proportional average abundances of bacterial populations and beta distances between communities in each day-10 fecal sample. Significant differences for the correlations were calculated using a Monte Carlo test with 999 iterations.

## Results

### TNF ablation results in diminished colitis

**Fig 1:**
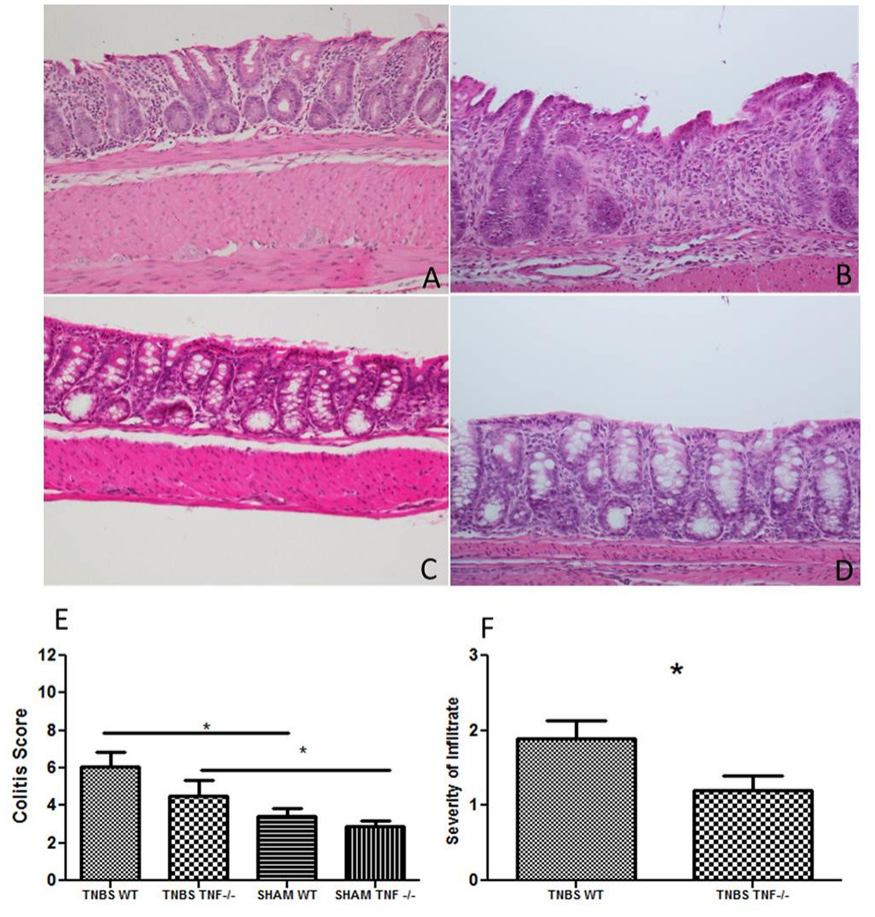
TNBS treatment induces significantly more inflammation than SHAM treatment in both WT and *Tnf* ^*-/-*^ mice and WT mice have a significantly more severe inflammatory infiltrate than *Tnf* ^*-/-*^ mice. Representative photomicrographs of the colons of TNBS treated *Tnf-/-* (A) and WT mice (B) and SHAM treated *Tnf-/-* (C) and WT mice (D). Histopathological semi-quantitative scores are presented as the mean score ± SE (E). Severity of infiltrate criterion is presented as the mean score ± SE (F). *, *p*.*05* (n = 10 mice per group)

There was significantly more severe disease in TNBS treated mice (Fig. 1A, B, and E). Sham treatment induced mild and mixed cellular inflammation in the distal colon without evidence of necrosis, erosions and ulceration (Fig. 1C and D). The inflammation was characterized by infiltration of neutrophils, lymphocytes and plasma cells and occasional necrosis and ulceration. Colitis scores account for inflammation (severity and distribution), necrosis and ulceration. When severity of inflammation was evaluated as a sole criterion, we found that there was significantly more severe inflammation in the TNBS treated WT mice than *Tnf* ^*-/-*^ mice (Fig 1F). These data indicate that the absence of TNF results in less severe TNBS-induced inflammation. Other clinical parameters of disease (body weights, rectal bleeding, colon shortening) did not differ significantly between TNBS treated WT and *Tnf* ^*-/-*^ mice. To further evaluate the role of TNF in driving inflammation in this model, we analyzed the microbiota of both genotypes of mice, before and after TNBS and SHAM treatment to determine if specific microbes or microbial communities contribute to the disease seen in WT mice.

### Deep sequencing indicates two phyla dominate mice gut communities

**Fig 2:**
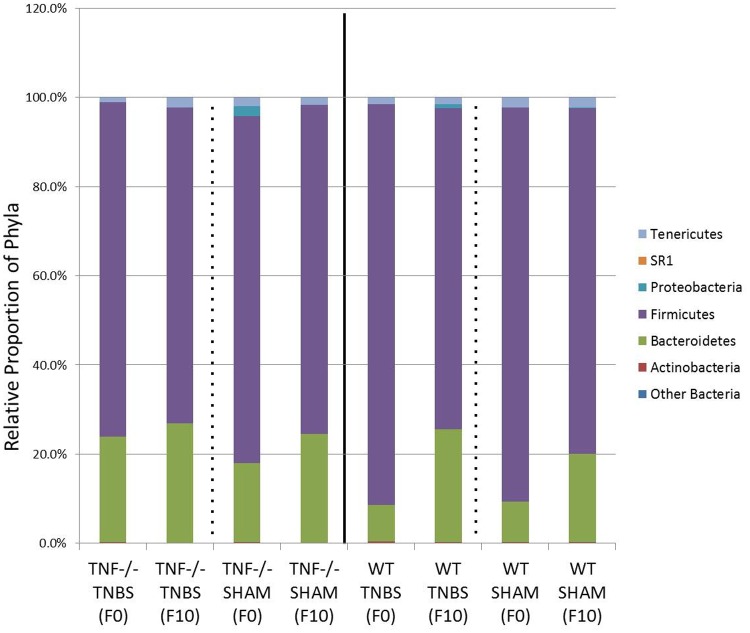
Bacteroidetes and Firmicutes are the predominant relative proportions of phyla in the feces of WT and *Tnf* ^*-/-*^ mice prior to colitis (F0) and post colitis (F10) in SHAM and TNBS treated mice. ANOVA indicates significant differences between Bacteroidetes and Firmicutes.

Illumina sequencing of the V3–4 region of the 16S rRNA gene of fecal bacterial community yielded a total of 5,484,879 paired end merged sequences after quality filtering and ranged from 30,330 to 119,346 sequences per sample with an average of 68,561 (± 17,910) sequences. Based on these numbers, all results reported herein use a subset of 30,330 sequences for each sample. More than 95% of the sequences in all mice at days 0 and 10 were classified into two phyla, Bacteroidetes and Firmicutes (Fig. 2). Other phyla present on average relative percentages per treatment that ranged from 0.001 to 2.36% were *Actinobacteria*, *Proteobacteria*, SR1 and *Tenericutes*. Taxonomic assignment of the sequences in all samples resulted in the identification of 50 genera, and an additional 39 taxa that were not classified to a known genus (S1 Table). Of these 89 taxa the majority of the communities were comprised of only 16 taxa that represented at least an average of 1% of the community in at least one treatment.

### Alpha and Beta diversity of communities differ significantly between WT and *Tnf* ^*-/-*^ mice

**Fig 3:**
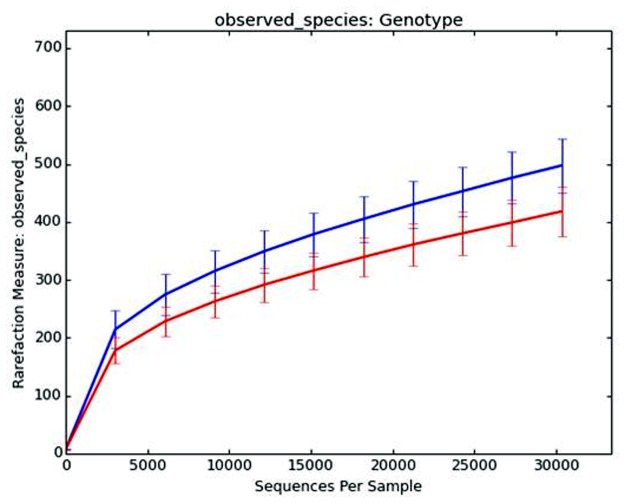
*Tnf* ^*-/-*^ mice had significantly less α-diversity (e.g. Observed Species) than WT mice. Differences significant (*p = 0*.*001*) using non-parametric t-test with 999 Monte Carlo permutations and Bonferroni correction. Data rarefied (10 iterations) to a maximum depth of 30330 reads per sample.

Alpha diversity is defined as the mean species diversity within a habitat—in this case diversity in each fecal sample [52]. Comparisons of the rarefied 30,330 sequences from each sample at days 0 and 10 indicated there were significant differences in community alpha diversity measures. A Good’s coverage average of 99.4% (range 99.1–99.7%) indicated there was sufficient community coverage using this dataset. There were significant differences (p = 0.001) between the WT and *Tnf* ^*-/-*^ mice for all the alpha diversity measures evaluated, the richness indices ChaoI (WT 1029.9 ± 130.5 vs *Tnf* ^*-/-*^ 879.1 ± 136.8) and observed species (WT 497.2 ±46.4 vs *Tnf* ^*-/-*^ 418.1 ± 42.2), and phylogenetic diversity (PD) whole-tree measure (WT 21.8 ± 1.4 vs *Tnf* ^*-/-*^ 19.0 ± 1.5) (Fig. 3 e.g., observed species). Differences were not significant between alpha diversity values of day 0 and 10 for each mouse genotype after TNBS or SHAM treatments.

**Fig 4:**
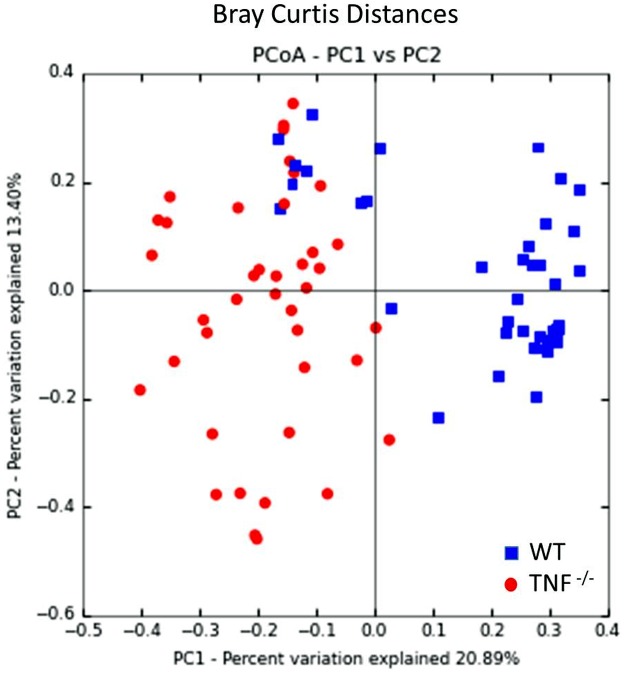
PCoA of β-diversity comparison using Bray Curtis distances revealed significant separation of microbial communities based on genotype. *p<0*.*00*1, using perMANOVA, additional analysis using PERMDISP indicates dispersion does not contributes significantly to these differences.

Beta diversity is defined as difference in diversity among habitats (e.g., among fecal samples); these differences can be determined using a variety of algorithms for distance measures [52]. All the beta diversity distance measures we tested indicated that there were host factors contributing to differences among gut microbial communities. Principal coordinate analysis (PCoA) of non-phylogenetic (binary Euclidean and Bray Curtis) and phylogenetic distances (weighted and unweighted Unifrac) illustrated the main separation of communities was by mouse genotype (Fig. 4, e.g., Bray Curtis distances) and perMANOVA indicated that these differences were significant (p = 0.001). There was also clustering within genotypes by TNBS treatment and sampling day (S1 Fig. e.g., Bray Curtis distances).

### TNF and Colitis induces significant differences in proportions of microbiota taxa

Comparisons of taxa proportions identified a number of groups that were possibly contributing to the differences in the community alpha and beta diversity measures. Significant differences were found between *Tnf* ^*-/-*^ and WT mice prior to colitis. At the phylum level significant differences were found between the Bacteroidetes (WT 8.6% ± 4.2% vs *Tnf* ^*-/-*^ 20.7% ± 16.1%) and Firmicutes (WT 89.1% ± 4.6% vs *Tnf* ^*-/-*^ 76.4% ± 15.8%) (n = 20 per genotype). Examination at the genus level indicated that within these phyla at day 0 there were three genera, *Ruminococcus*, *Turicibacter* and *Anaerovorax*, and five groups that have yet to be assigned to a genus that differed significantly (p < 0.05) (Table 1). There were also significant differences in taxa in healthy and colitic mice that were dependent and independent of TNF (Tables 2 and 3). The relative proportion of *Lactobacillus* decreased significantly from about 15% at day 0 to about 5% at day 10 in TNBS treated WT mice (Table 2). In the *Tnf* ^*-/-*^ mice significant differences in four taxa after TNBS and four other taxa after SHAM treatment but know of these taxa could be classified to any known genera (Table 3). There were significant differences in microbial communities between mice genotypes on day 0 before treatments were administered, therefore comparisons were also made between differences in the change of genera proportions from day 0 to day 10. In the WT mice three taxa differed significantly between day 0 and 10 (Table 4), most notably was the decrease in the genus *Turicibacter* after TNBS treatment. In the *Tnf* ^*-/-*^mice only the “Other” Ruminococcaceae group differed significantly (p = 0.01), -0.53 ± 1.28% (SHAM) vs 0.58 ± 0.52% (TNBS).

**Table 1:** Relative proportion (%) of bacterial genera that significantly differed between the two mouse genotypes on day 0.

Phylum	Class	Family	Genus	TNF-/-	WT	P
Bacteroidetes	Bacteroidia	S24–7	Unclassified	20.69 ±16.14	8.60 ±4.25	0.050
Firmicutes	Bacilli	Other	Other	0.00 ±0.00	0.05 ±0.08	0.000
Firmicutes	Bacilli	Turicibacteraceae	*Turicibacter*	0.40 ±1.08	6.09 ±8.28	0.002
Firmicutes	Clostridia	Other	Other	0.01 ±0.01	0.03 ±0.02	0.001
Firmicutes	Clostridia	Clostridiaceae	Unclassified	1.77 ±2.97	4.34 ±4.61	0.035
Firmicutes	Clostridia	Ruminococcaceae	Unclassified	1.54 ±0.74	3.66 ±1.54	0.000
Firmicutes	Clostridia	Ruminococcaceae	*Ruminococcus*	2.63 ±1.87	1.37 ±0.66	0.037
Firmicutes	Clostridia	[Mogibacteriaceae]	*Anaerovorax*	0.01 ±0.01	0.00 ±0.00	0.002

n = 20 for each genotype. Significance tested using Mann Whitney U with Bonferroni correction.

“Unclassified” are taxa in the families listed that are yet to be classified with a genus name. “Other” is taxa that cannot be clearly assigned to a reference group in the Greengenes data set (version 13_5).

**Table 2:** Relative proportions of significantly different bacterial genera in WT mice.

			TNBS		SHAM	
Phylum	Family	Genus	F0	F10	F0	F10
Bacteroidetes	S24–7	Unclassified	8.11 ±4.12^a^	25.23 ±18.44^b^	9.10 ±4.54^a^	19.76 ±11.29^b^
Firmicutes	Lactobacillaceae	*Lactobacillus*	15.47 ±13.98^a^	5.04 ±4.89^b^	9.13 ±4.59^a^	7.29 ±6.26^ab^
Firmicutes	Turicibacteraceae	*Turicibacter*	8.71 ±9.85^ab^	6.10 ±3.75^b^	3.46 ±5.69^a^	9.62 ±6.18^b^
Firmicutes	Ruminococcaceae	Other	0.70 ±0.55^a^	0.52 ±0.30^a^	0.73 ±0.21^a^	0.47 ±0.23^b^
Firmicutes	Ruminococcaceae	Unclassified	3.35 ±1.79^ab^	3.03 ±3.66^b^	3.97 ±1.27^a^	2.48 ±0.94^b^
Firmicutes	[Mogibacteriaceae]	Unclassified	0.01 ±0.02^a^	0.05 ±0.04^b^	0.01 ±0.01^a^	0.04 ±0.03^b^

Superscript letters across rows denote significant difference at P<0.05 determined using Mann Whitney U test.

“Unclassified” are taxa in the families listed that are yet to be classified with a genus name. “Other” are taxa that cannot be clearly assigned to a reference group in the Greengenes data set (version 13_5).

**Table 3:** Relative proportions of significantly different bacterial genera in *Tnf* ^*-/-*^ mice.

			TNBS		SHAM	
Phylum	Family	Genus	F0	F10	F0	F10
Firmicutes	Planococcaceae	Unclassified	0.38 ±1.03^a^	0.00 ±0.00^a^	0.72 ±1.95^b^	0.00 ±0.00^a^
Firmicutes	Other Clostridiales	Other	0.01 ±0.01^ab^	0.01 ±0.01^ab^	0.003 ±0.00^a^	0.02 ±0.02^b^
Firmicutes	Clostridiaceae	Other	0.004 ±0.01^ab^	0.002 ±0.00^ab^	0.00 ±0.00^a^	0.01 ±0.01^b^
Firmicutes	Clostridiaceae	Unclassified	0.68 ±1.47^a^	1.51 ±1.83^b^	2.85 ±3.72^b^	3.49 ±4.34^b^
Firmicutes	Lachnospiraceae	Other	0.10 ±0.10^a^	0.69 ±0.80^b^	0.15 ±0.18^a^	0.37 ±0.49^a^
Firmicutes	Ruminococcaceae	Other	0.72 ±0.63^a^	1.30 ±0.49^b^	1.39 ±1.23^ab^	0.86 ±0.51^ab^
Firmicutes	Ruminococcaceae	Unclassified	1.31 ±0.50^a^	2.08 ±0.67^b^	1.77 ±0.88^ab^	1.75 ±0.61^ab^
Proteobacteria	Enterobacteriaceae	Unclassified	0.00 ±0.00^a^	0.00 ±0.00^a^	0.00 ±0.00^a^	0.01 ±0.01^b^

Superscript letters across rows denote significant difference at P<0.05 determined using Mann Whitney U test.

“Unclassified” are taxa in the families listed that are yet to be classified with a genus name. “Other” are taxa that cannot be clearly assigned to a reference group in the Greengenes data set (version 13_5).

**Table 4:** Change from day 0 to day 10 of proportions of significantly different bacterial genera in WT mice.

Phylum	Family	Genus	TNBS	SHAM	P
Firmicutes	Planococcaceae	Unclassified	0.03 ± 0.08	-0.02 ± 0.04	0.018
Firmicutes	Turicibacteraceae	*Turicibacter*	-2.61 ± 8.79	6.16 ± 6.17	0.028
Firmicutes	Peptostreptococcaceae	Unclassified	0.03 ± 0.10	0.00 ± 0.02	0.035

Proportional changes were calculated by subtracting F0 from F10 values. Significant difference at P<0.05 determined using Mann Whitney U test. “Unclassified” are taxa in the families listed that are yet to be classified at the genus level.

### Bacterial communities and colitis criteria were significantly correlated

**Fig 5:**
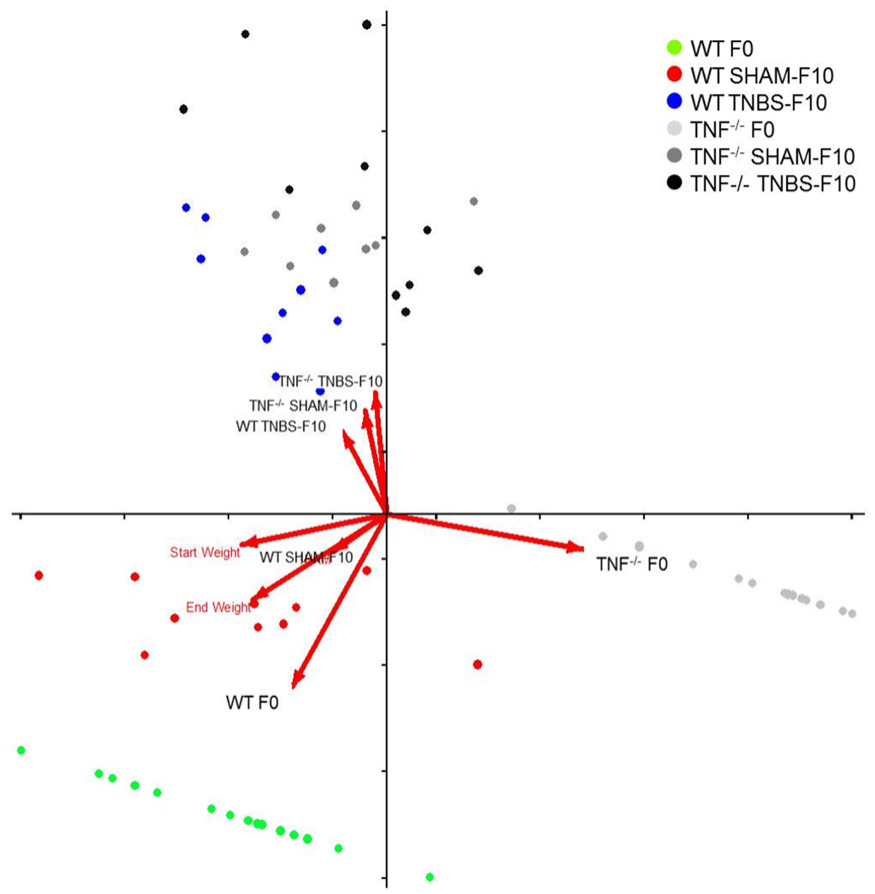
Canonical correspondence analysis (CCA) of fecal bacterial composition, mouse genotype, treatment, sampling date and weight of mice. Fecal sample collected from WT and *Tnf* ^*-/-*^ mice treated with TNBS or SHAM at the beginning (F0) versus end (F10) of acute treatment. Variation explained in horizontal axis is 7.7% and the vertical axis is 2.7%.

**Fig 6:**
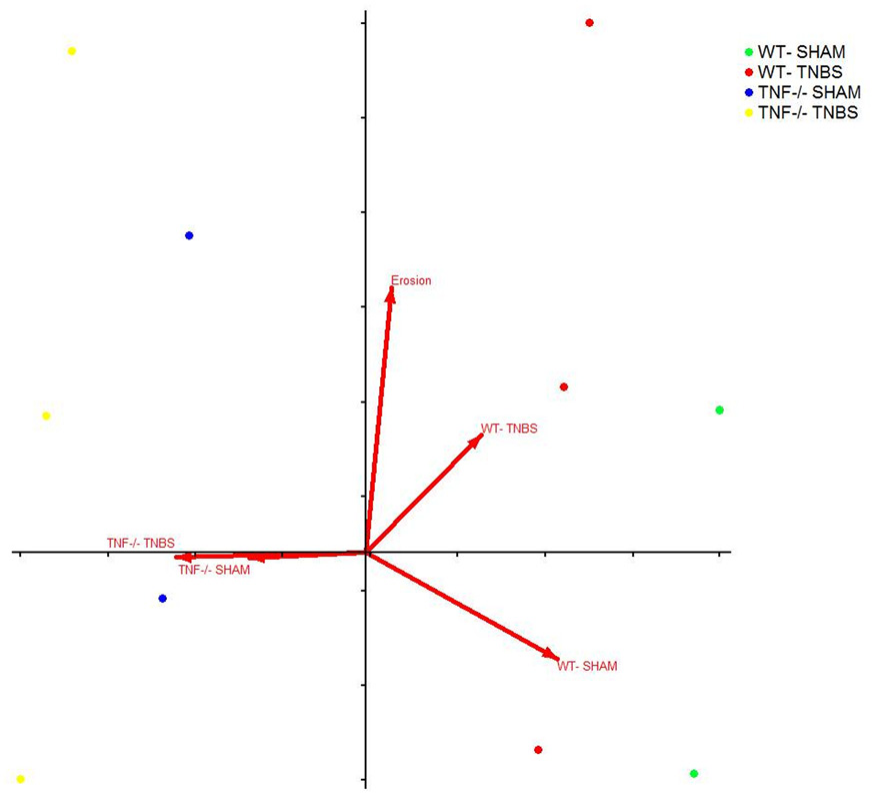
Canonical correspondence analysis (CCA) of fecal bacterial composition, mouse genotype, treatment, and histopathology criterion of mice on day 10 revealed more close associations between genotype and erosions in TNBS treated WT mice. Variation explained in horizontal axis is 7.9% and the vertical axis is 6.8%.

Canonical correspondence analysis (CCA) indicated that the variation in bacterial community composition, treatment and some mouse traits had significant correlations. Factors that significantly corresponded (p<0.05) with the microbial communities were mice genotype, starting weight and ending weights (Fig. 5). The relative orientation of arrows in the plot illustrates the direction of maximum difference of corresponding factors and the arrow length depicts the magnitude of the differences. Arrows in the lower left quadrant indicates correspondence between mice weight that was greater in microbial communities in samples from WT mice at day 0 and SHAM treated WT mice on day 10. In a perpendicular orientation in the upper right quadrant is an arrow for the *Tnf* ^*-/-*^ mice on day 0, confirming that *Tnf* ^*-/-*^ mice weigh less than WT mice at day 0. The arrows along the upper y-axis indicate there is correspondence between the microbial communities of SHAM and TNBS treated *Tnf* ^*-/-*^mice on day 10. Since colitis scores were taken only on day 10, another CCA was used to determine correspondence with day 10 microbial communities samples (Fig. 6). Differences in each colitis criteria and the colitis score as a whole (along upper y-axis) were found to correspond closest to the microbial communities at day 10 in WT TNBS treated mice (upper right quadrant) and furthest from WT SHAM mice. Whereas, in opposite orientation to WT-TNBS mice along the x-axis are the arrows for *Tnf* ^*-/-*^mice treated with TNBS or SHAM, indicating that it is the lack of TNF production that is protective against ulceration formation.

## Discussion

In this study, we evaluated how TNF ablation affects acute colitis, and how colitis correlates with TNF dependent alterations in the microbiota. As expected, we found that TNF promotes acute TNBS colitis, and CCA indicated colitis scores corresponded with alterations in the colonic microbiota. The decreased inflammation in TNF- mice is consistent with previous reports [53,54]. Similarly, treatment of mice with an anti-TNF monoclonal antibody also caused decreased mucosal cellular infiltration and down regulation of pro-inflammatory cytokine levels [55,56]. Anti-TNF antibody administration resulted in significant apoptosis of macrophages [55] in the lamina propria or of enterocytes [56], which was considered to be the most likely mechanism of disease remission. We believe that these findings support the accepted theory that TNF is a master regulator and is critical in acute inflammatory responses and that the lack of TNF production or signaling resulted in decreased colitis, which otherwise would likely have proceeded uninhibitedly. Additionally, TNF has been shown to inhibit the repair of the gut epithelium via increasing the number of apoptotic cells in both mice and humans [57–59], which contributes to the inflammation associated with the translocation of bacteria. Here, we expanded upon these studies and included investigations of the effect of TNF-depletion on the intestinal microbiota.

TNBS treatment of WT or *Tnf* ^*-/-*^ mice did not decrease alpha diversity significantly after 10 days but WT mice developed more severe colitis. Other studies have found a decrease in alpha diversity associated with IBD in people [24] but a10-day acute study may not be sufficient to see a statistically significant change in alpha diversity. A notable observation is that alpha diversity was lower in *Tnf* ^*-/-*^ mice than in WT mice before any treatment but *Tnf* ^*-/-*^ mice developed less severe colitis. It is possible that the gene mutation is sufficient for colitis protection and the microbiota are not playing a major role in the pathology or the necessary microbiota are still present for protection. In contrast, compared to WT mice alpha diversity was higher in mice lacking the Tata Element Modulatory Factor (TMF/ARA160) and the microbiota were playing a role in protection from chronic colitis [60]. A longer study is needed to determine if *Tnf* ^*-/-*^ mice have chronic protection from colitis and despite the lower diversity if the microbiota play a role in protection.

Interestingly, *Tnf* ^*-/-*^ mice are generally smaller than their WT counterparts and, alpha and beta diversity comparisons using CCA indicated that the smaller size corresponded with differences in the microbial communities. Further studies are needed to determine if the differences in microbiota community composition are a contributing cause of the lower body weight of *Tnf* ^*-/-*^ mice or if other factors associated with TNF ablation causes lower body weight, which then leads to the difference in the colonic communities. The lower weight and lower microbial diversity observed in the *Tnf* ^*-/-*^ mice may contribute to more detrimental pathology when the disease is chronic or cause secondary health problems.

Beta diversity comparisons revealed that the most significant difference in communities was due to mouse genotype. At the phylum level this difference was likely due to the higher Firmicutes to Bacteroidetes ratio in WT vs *Tnf* ^*-/-*^ mice. This differs from a study that used anti-TNF-α to inhibit TNF production in mice in which the Firmicutes to Bacteroidetes ratio decrease but no information was given if this difference was significant [61]. This suggests that the method of TNF ablation can affect the microbial community differently but both approaches provided colitis protection. At the genus level there were significant differences in a number of taxa between WT and *Tnf* ^*-/-*^ mice but the roles of many of them are currently not known. However, there have been reports on *Ruminococcus*, *Turicibacter*, and unclassified S24–7. *Ruminococcus* is believed to be a beneficial microbe that can ferment resistant starches [62]. It was found in significantly higher proportion in *Tnf* ^*-/-*^ compared to WT mice potentially contributing to protection from colitis. There was a significantly greater proportion of *Turicibacter* in WT compared to *Tnf* ^*-/-*^ mice both prior to and after colitis induction, suggesting that its presence may be related to TNF expression. Little is known about the role of *Turicibacter* in IBD. However, one study found an increase in *Turicibacter* in mice with depletion of CD8+ T cells [63], while another isolated this bacterium from the serum of an acutely ill patient [64]. These seemingly contradicting results warrant further study of how this bacterium is potentially involved in IBD. There was a significantly higher proportion of S24–7, an unclassified group of bacteria in the phylum Bacteroidetes, at day 0 in *Tnf* ^*-/-*^ mice compared to WT mice. Recently, this group of bacteria has been found to be associated with remission of colitis in mice [65]. This may explain our finding of greater proportions of these bacteria in *Tnf* ^*-/-*^ mice, which develop less colitis than WT mice. A bacterium that may be playing a protective role in WT mice is *Lactobacillus*. There was a significant decrease in *Lactobacillus* in WT mice after TNBS treatment. This genus is known to be “beneficial” and is extensively used as a probiotic in a variety of diseases, including gastrointestinal diseases [66]. The decrease in this bacterium in mice with greater colitis supports its role as an organism that promotes intestinal health.

We describe here for the first time, the effects of TNF ablation on acute TNBS colitis and the microbiota. Our data demonstrate a pathogenic role for TNF in acute TNBS colitis and importantly, we show for the first time how inflammation (and TNF production) is associated with significant differences in the microbiota. These microbiota data support our general conclusion that the strongest factor contributing to the microbiome alterations, and subsequently, inflammation, in this model is TNF production. These findings suggest that combinatory therapy including inhibition of TNF and the target altering specific microbial communities (e.g. via probiotic therapy) may prove to be a beneficial therapeutic approach for IBD patients.

## Supporting Information

S1 Fig PCoA of Bray Curtis distances between samples labeled according to the mouse genotype, treatment and day of sample collection. Significant differences between these groups (*p<0*.*001*) using perMANOVA, but additional analysis using PERMDISP indicates dispersion is significant, mainly due to the relatively tight cluster of WT-SHAM F0 samples compared to greater dispersion of the other groups.
(TIFF)

S1 Table Mean proportion of taxa per treatment calculated using an equivalent number of reads per sample (XLSX)
