# Correction: Ablation of Tumor Necrosis Factor Is Associated with Decreased Inflammation and Alterations of the Microbiota in a Mouse Model of Inflammatory Bowel Disease

**DOI:** 10.1371/journal.pone.0125309

**Published:** 2015-04-10

**Authors:** Yava L. Jones-Hall, Ariangela Kozik, Cindy Nakatsu

There are errors in the third sentence of the Abstract. The third sentence should instead be two separate sentences, which should read as follows: However, some patients are non-responsive to this therapy, eventually become refractory to therapy, or may develop harmful side-effects. Alterations in the microbiota that are associated with the lack of TNF could be a contributing cause of the therapeutic insufficiency seen in some patients.
