# Supplementary figures and images for: Ablation of Tumor Necrosis Factor Is Associated with Decreased Inflammation and Alterations of the Microbiota in a Mouse Model of Inflammatory Bowel Disease
Source: PLoS One. 2015 Mar 16;10(3):e0119441. doi: 10.1371/journal.pone.0119441 (PMC4361568; doi:10.1371/journal.pone.0119441)

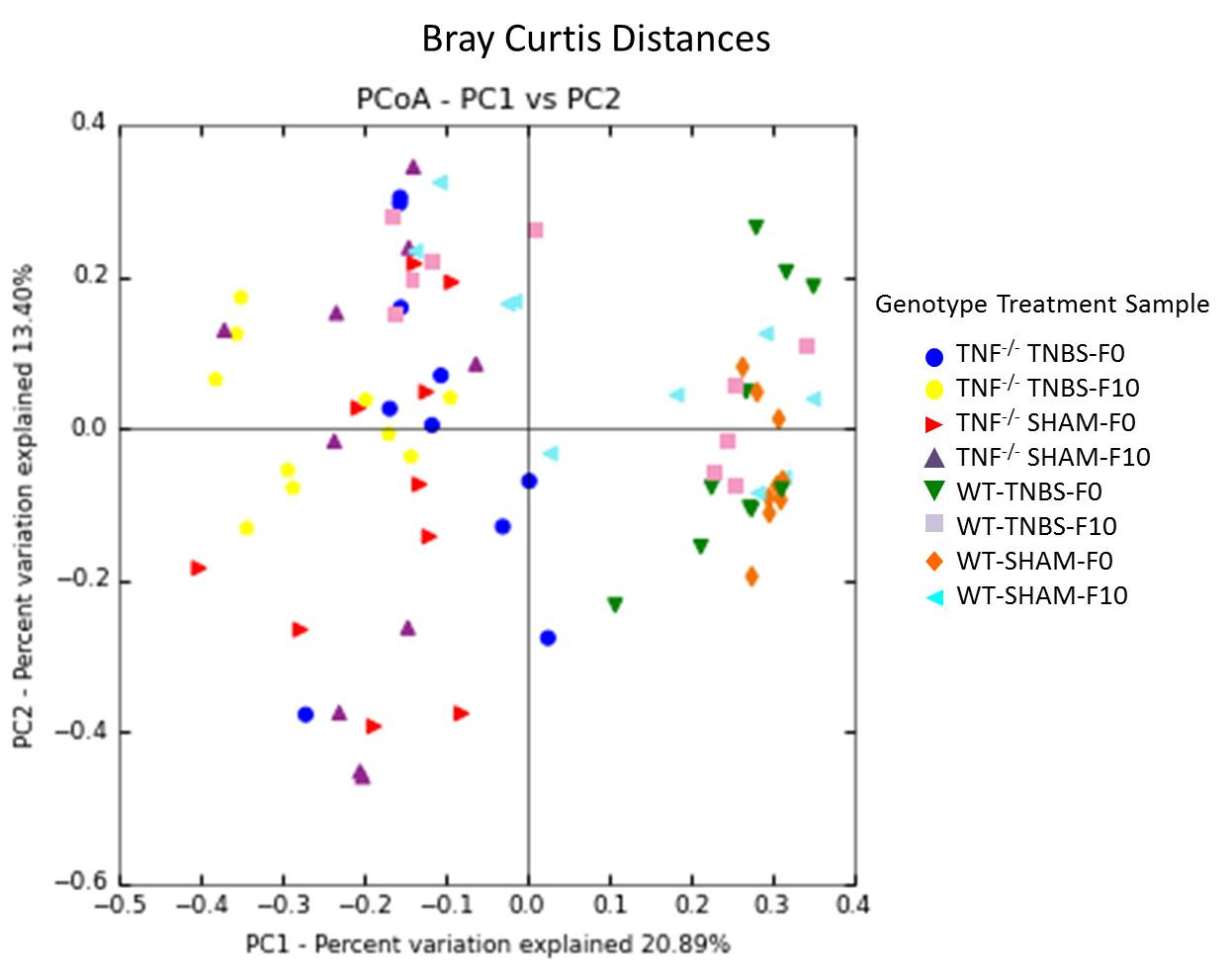

Supplement: S1 Fig — Significant differences between these groups (p<0.001) using perMANOVA, but additional analysis using PERMDISP indicates dispersion is significant, mainly due to the relatively tight cluster of WT-SHAM F0 samples compared to greater dispersion of the other groups. (TIFF) [file pone.0119441.s001.tiff]
